# Emergence of *Trichophyton tonsurans*—A Retrospective Multicentre Study of the Dermatophyte Spectrum in Germany

**DOI:** 10.1111/myc.70053

**Published:** 2025-04-08

**Authors:** Julia Felicitas Pilz, Martin Schaller, Martin Köberle, Alexandra Lorz, Avend Bamarni, Sebastian Sitaru, Franziska Schauer, Hans Peter Seidl, Tilo Biedermann, Alexander Zink, Kilian Eyerich, Anna Caroline Pilz

**Affiliations:** ^1^ Department of Dermatology and Allergy Technical University of Munich, School of Medicine and Health Munich Germany; ^2^ Department of Dermatology Eberhard Karls University of Tübingen Tübingen Germany; ^3^ Department of Dermatology and Venereology Medical Center, University of Freiburg Freiburg Germany; ^4^ Christine Kühne‐Center for Allergy Research and Education, Medicine Campus Davos Davos Switzerland; ^5^ Division of Dermatology and Venereology, Department of Medicine Solna Karolinska Institute Stockholm Sweden

**Keywords:** dermatophytes, epidemiology, fungal skin infections, Germany, *tinea capitis*, *tinea corporis*, *Trichophyton tonsurans*

## Abstract

**Background:**

In mid‐2024, German media reported increasing fungal infections by *Trichophyton tonsurans* linked to visits to barbershops. However, epidemiological data confirming a rise in *tinea capitis* and *tinea corporis* due to *Trichophyton tonsurans* are lacking.

**Objectives:**

This study assesses dermatophyte species and clinical types of infections in German university hospitals in 2018 and 2023.

**Patients/Methods:**

This retrospective, multicentre study analyses mycological culture results from three Departments of Dermatology in Freiburg, Tübingen and Munich. The dermatophyte, along with the sampled body site, age and gender of the affected patient, was recorded.

**Results:**

1915 patients (male: 66.1%; mean age: 50 ± 24 years) with a dermatophyte‐positive culture were identified. The most common dermatophyte was *Trichophyton rubrum* (2018: 78.7%; 2023: 66.3%) with *tinea pedis* and *tinea unguium* being the most prevalent types of infection. An increase in *tinea corporis* and *tinea capitis was observed*, with *tinea capitis* doubling from 4.3% to 9.3%. In 2023, *Trichophyton tonsurans* emerged as the prevailing dermatophyte (67.6%) in *tinea capitis* and as the second most frequent agent in *tinea corporis* (26.3%). This dominance of *Trichophyton tonsurans* was consistently observed across all three study centres. *Trichophyton tonsurans* affected patients presented a median age of 18 years in 2023 (vs. 9 years in 2018) and an amplified imbalance towards the male gender.

**Conclusions:**

The pathogen spectrum and infection patterns have changed in Germany due to the increase of *Trichophyton tonsurans* infections. Intensified screening and hygiene measures, as well as adaptation of initial empiric treatment of *tinea capitis,* should be considered.

## 1 Introduction

Dermatomycoses, fungal infections of the skin, nails and hair, affect approximately 20%–25% of all people worldwide [1] and are mainly caused by dermatophytes, followed by yeasts such as *Candida* species and rarely by molds [2].

On a global scale, most dermatophytoses arise from *Trichophyton* (*T*.) species, the majority by *T. rubrum* [ 3], which is an anthropophilic agent capable of infecting all body sites. However, the prevalence of other causative species varies considerably depending on the country/region analysed, as well as body site, gender, age, socioeconomic status and comorbidities [4]. For example, in *tinea capitis* worldwide, *T. violaceum* and *T. tonsurans* are the leading anthropophilic agents, and *Microsporum* (*M*.) *canis* is the predominant zoophilic agent, but there are great regional differences. In North Africa, *T. violaceum* and *M. canis* ; in South Africa, *T. violaceum* and *M. audouinii*; in the United States, *T. tonsurans* and in Thailand, *T. rubrum* and *T. mentagrophytes* are the most frequent agents. Additionally, the prevalence of causative organisms varies constantly due to migration, globalisation as well as changes in climate, transmission routes or lifestyle [4, 5].

In Germany, epidemiological data on the pathogen spectrum of dermatophytoses are generally scarce and, when available, often focus on single centres and/or single diagnoses or time spans. In Würzburg, Germany, from 1990 to 2014, an increase in the total number of identified species in *tinea capitis* from 8 to 11 was described, with *M. canis* being the most frequent causative agent [6]. In Dessau, Göttingen and Jena, dermatophyte cultures from 01/2014 to 12/2016 were analysed, identifying *M. canis* , *T. mentagrophytes* and *T. benhamiae* as the main pathogens of *tinea capitis*, and *T. rubrum* as the predominant pathogen in *tinea corporis*, *manuum*, *pedis* and *unguium* [7]. In mid‐2024, several popular German newspapers, TV stations and social media content creators reported an increase in *tinea capitis* caused by *T. tonsurans*, associated with visits to barbershops [8, 9, 10, 11]. In Germany, there are currently no epidemiological data identifying *T. tonsurans* as one of the major players in *tinea capitis* and *corporis* on a national scale. Mostly local outbreaks, especially in relation to wrestling and/or kindergartens, have been observed [12, 13, 14]. However, two single‐centre studies in Munich recently identified an increase in *T. tonsurans* infections from 2019–2022 and 2014–2019, respectively [15, 16]. In addition, in Kiel, the public health department identified *T. tonsurans* in three out of 10 samples tested in a barbershop following up on a complaint from a citizen with *tinea capitis* [17].

The present study aims to analyse the dermatophyte spectrum at three large university hospitals in southern Germany in 2018 and 2023, comparing species and types of infection.

## 2 Materials and Methods

### 2.1 Study Design

This retrospective, multicentre study analysed all dermatophyte‐positive cultures from the Departments of Dermatology at three university hospitals—the University Medical Center Freiburg, the Tübingen University Hospital and the University Hospital of the Technical University of Munich—in southern Germany between January and December 2018 and 2023. The study was conducted in accordance with the Declaration of Helsinki and was approved by the Ethics Committee of the University Medical Center Freiburg (reference number 24‐1321_1‐S1‐retro) as well as by the ethics committees at all participating sites.

### 2.2 Data Collection and Analysis

All culture samples included in this study were obtained during routine diagnostic procedures. The data collected included the causative dermatophyte species identified in the cultures, along with the patient's age, gender and the specific body area from which the sample was taken.

Types of infection were classified as *tinea capitis, tinea faciei, tinea corporis, tinea manus, tinea pedis* or *tinea unguium*. *Tinea capitis* was defined as involvement of the scalp, eyebrows or beard, while *tinea faciei* referred to infections of the face. *Tinea corporis* included infections of the neck, trunk, limbs and inguinal region, whereas *tinea manus* and *tinea pedis* were restricted to the hands and feet, respectively. *Tinea unguium* was defined as infection of the fingernails and/or toenails. The combined analysis of *tinea capitis* plus *tinea corporis* included all patients with *tinea capitis* and/or *tinea corporis*. If multiple body areas of the same patient were affected, each one was recorded. In cases of infections caused by more than one dermatophyte species, all identified species were documented. For repeated detection of the same dermatophyte in the same body area within a single year, only one instance was counted.

### 2.3 Statistical Analyses

Epidemiological data were analysed using descriptive statistics including mean, standard deviation (SD), median, interquartile range (IQR), absolute numbers and proportions. Data processing and figure generation were performed with Microsoft Excel and Microsoft PowerPoint.

## 3 Results

### 3.1 Study Cohort Characteristics

Combining data from all three study centres (Freiburg, Tübingen and Munich) for the years 2018 and 2023, a total of 1915 patients with a dermatophyte‐positive culture were identified. Of these, 1266 (66.1%) were male, and the mean age was 50 ± 24 years with 261 (13.6%) being younger than 18 years (Table 1).

**TABLE 1:** Characteristics of the study cohort (SD = standard deviation).

Characteristic	*n* (%)
Patients with dermatophyte‐positive culture	1915 (100)
Freiburg	477 (24.9)
Tübingen	890 (46.5)
Munich	548 (28.6)
Male gender	1266 (66.1)
Age in years, mean ± SD	49.9 ± 24.334
< 18 years	261 (13.6)
≥ 18 years	1654 (86.4)

Abbreviation: *n*, number.

### 3.2 Causative Agents and Types of Infection of Dermatophytoses in Southern Germany in 2018 and 2023

**FIGURE 1:**
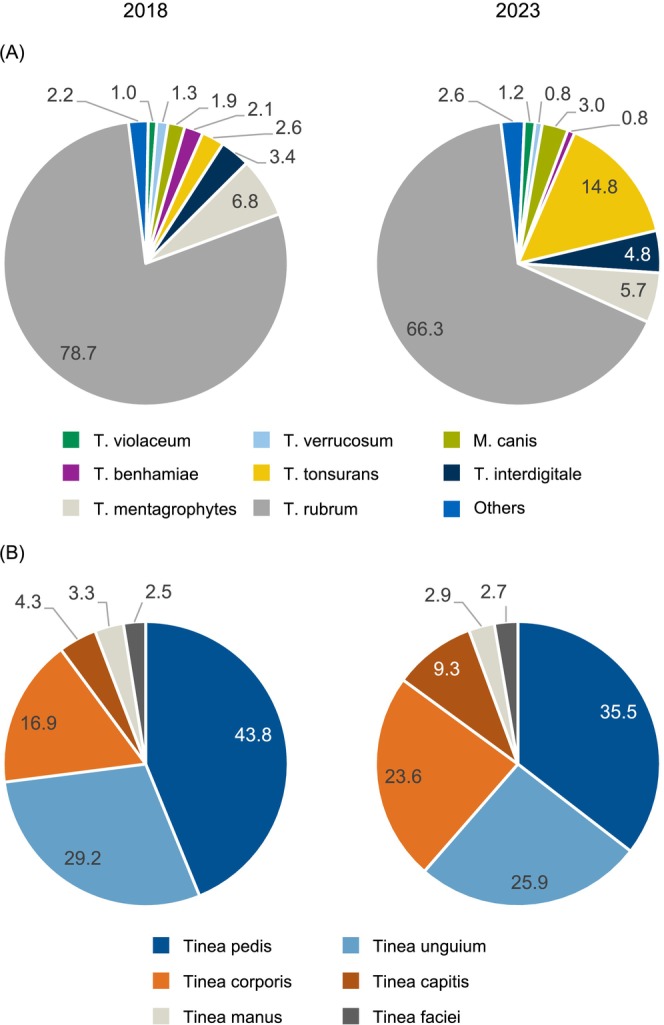
Causative agents and types of infection of dermatophytoses in 2018 and 2023. Distribution of causative agents (A) and types of infection (B) of dermatophytoses of all three study centres in 2018 (left) and 2023 (right). Percentages for each category are represented in the pie charts, with colours indicating the different categories. All dermatophytes with an absolute number ≥ 7 were categorised as ‘Others’ (Table S1).

In 2018 and 2023, *T. rubrum* was the most prevalent dermatophyte, accounting for 78.7% (739/939) and 66.3% (651/982) of all cases, respectively. In 2018, the four most common causative agents were *T. rubrum* , *T. mentagrophytes* (6.8%; 64/939), *T. interdigitale* (3.4%; 32/939) and *T. tonsurans* (2.6%; 24/939). By 2023, this distribution had shifted to *T. rubrum* , *T. tonsurans* (14.8%; 145/982), *T. mentagrophytes* (5.7%; 56/982) and *T. interdigitale* (4.8%; 47/982). Thus, *T. tonsurans* showed a 5.7‐fold increase. The proportions of *T. mentagrophytes* and *T. interdigitale* remained stable between 2018 and 2023, while *T. rubrum* exhibited a decrease of 12.4 percentage points. All other dermatophytes accounted for 3.0% or less of cases in both years (Figure 1A, Table S1).

Regarding types of infection, *tinea pedis* and *tinea unguium* were the most common dermatophytoses diagnosed in both 2018 and 2023. However, the proportion of *tinea pedis* decreased from 43.8% (454/1036) in 2018 to 35.5% (387/1091) in 2023, while the proportion of *tinea unguium* remained relatively stable, at 29.2% (302/1036) and 25.9% (283/1091). In contrast, the number of patients diagnosed with *tinea capitis* rose from 4.3% (45/1036) in 2018 to 9.3% (102/1091) in 2023, marking a 2.2‐fold increase, and the proportion of *tinea corporis* increased from 16.9% (175/1036) in 2018 to 23.6% (258/1091) in 2023 (Figure 1B).

### 3.3 Causative Agents of *Tinea Pedis, Tinea Unguium, Tinea Corporis* and *Tinea Capitis* in Southern Germany in 2018 and 2023

Having observed a shift in the distribution of dermatophytes, with the most notable change being an increase of *T. tonsurans*, the distribution of causative agents was further analysed for the four most common types of infection: *tinea pedis*, *tinea unguium*, *tinea corporis* and *tinea capitis*.

**FIGURE 2:**
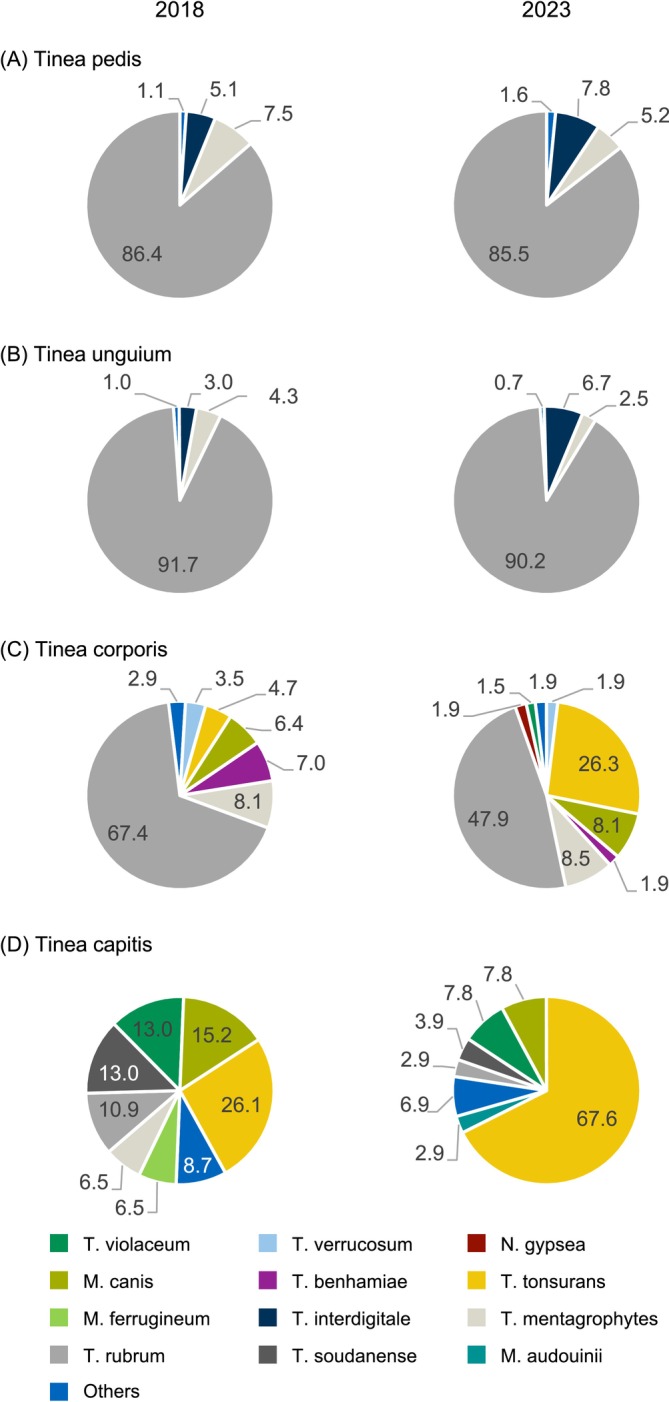
Causative agents of *tinea pedis, tinea unguium, tinea corporis* and *tinea capitis* in 2018 and 2023. Distribution of causative agents of *tinea pedis* (A), *tinea unguium* (B), *tinea corporis* (C) and *tinea capitis* (D) of all three study centres in 2018 (left) and 2023 (right). Percentages for each category are represented in the pie charts, with colours indicating the different categories. All dermatophytes with an absolute number ≥ 2 were categorised as ‘Others’ (Table S2).

The distribution of causative agents for *tinea pedis* and *tinea unguium* remained highly consistent between 2018 and 2023. The three most prevalent pathogens for both conditions were *T. rubrum* , *T. mentagrophytes* and *T. interdigitale*, with *T. rubrum* being by far the predominant pathogen (Figure 2A,B; Table S2). *T. rubrum* also was the leading causative organism in *tinea corporis* in both years; however, its proportion declined from 67.4% (116/172) in 2018 to 47.9% (124/259) in 2023. Here, *T. tonsurans* increased from 4.7% (8/172) to 26.3% (68/259). The shares of *T. mentagrophytes* and *M. canis* remained consistent, and *T. benhamiae* declined from 7.0% (12/172) to 1.9% (5/259) in 2023 (Figure 2C).

The distribution of pathogens causing *tinea capitis* was rather evenly spread across different species in 2018. *T. tonsurans*, *M. canis* , *T. soudanense* and *T. violaceum* each accounted for 13%–26%. By 2023, the proportion of *T. tonsurans* had increased 2.6‐fold, rising from 26.1% (12/46) to 67.6% (69/102) of all *tinea capitis* cases. The following most common pathogens in 2023 were *M. canis* and *T. violaceum* , each accounting for 7.8% (8/102) of cases (Figure 2D).

### 3.4 Causative Agents of *Tinea Corporis* Plus *Tinea Capitis* in Freiburg, Tübingen and Munich in 2018 and 2023

We next asked whether the trend of increased *T. tonsurans* infections in *tinea corporis* and *tinea capitis* was a consistent observation in all three study centres. Therefore, *tinea corporis* and tinea *capitis* cases were combined and analysed for each of the three sites separately.

**FIGURE 3:**
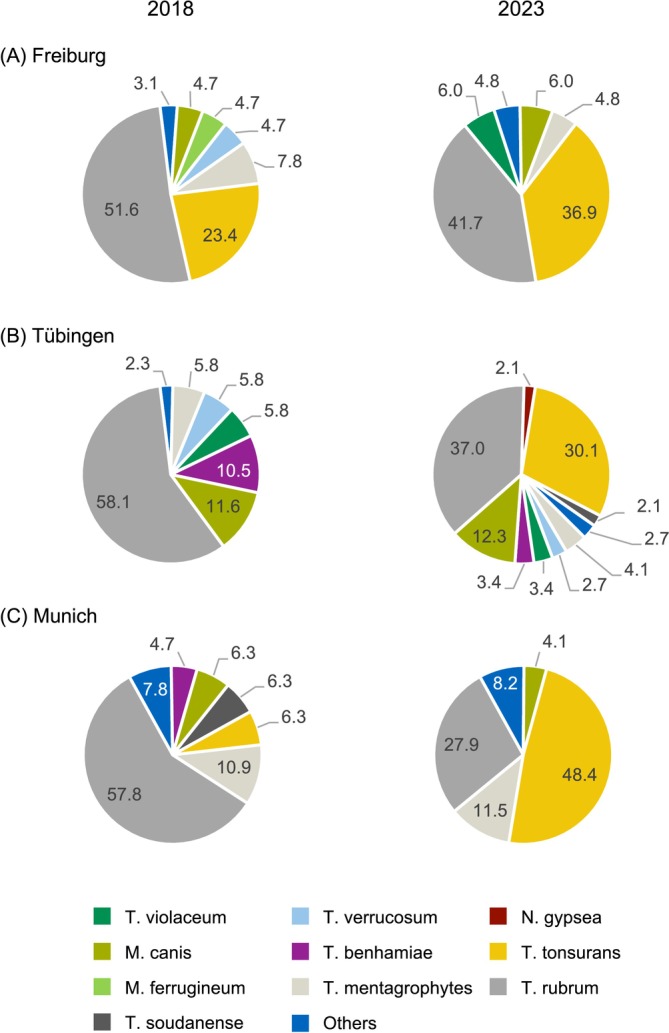
Causative agents of *tinea corporis* plus *tinea capitis* in Freiburg, Tübingen and Munich in 2018 and 2023. The distribution of causative agents of *tinea corporis* plus *tinea capitis* (combined) categorised by the study centres Freiburg (A), Tübingen (B) and Munich (C) for the years 2018 (left) and 2023 (right). Percentages for each category are represented in pie charts, with colours indicating the different categories. All dermatophytes with an absolute number ≥ 2 were categorised as ‘Others’ (Table S3).

In Freiburg, *T. tonsurans* was already the second most frequent dermatophyte (23.4%; 15/64), following *T. rubrum* (51.6%; 33/64), in *tinea corporis* plus *tinea capitis* in 2018. Nevertheless, the proportion of *T. tonsurans* increased by 12.9 percentage points to 36.9% (31/84) in 2023, while *T. rubrum* declined by 9.9 percentage points to 41.7% (35/84; Figure 3A, Table S3).

In Tübingen, *T. tonsurans*, which had not been detected at all in 2018, emerged as the second most frequent pathogen in 2023, representing 30.1% (44/146) of cases nearly matching *T. rubrum* in frequency (37.0%, 54/146; Figure 3B).

In Munich in 2018, the primary pathogen causing *tinea corporis* plus *tinea capitis* was *T. rubrum* (57.8%; 37/64), followed by *T. mentagrophytes* (10.9%; 7/64). By 2023, the leading pathogen had shifted from *T. rubrum* to *T. tonsurans*. *T. tonsurans*, which represented 6.3% (4/64) of cases in 2018, experienced a 7.7‐fold increase, rising to 48.4% (59/122) (Figure 3C).

### 3.5 Gender and Age Distribution for *T. tonsurans*‐Induced *Tinea Corporis* and *Tinea Capitis* in Southern Germany in 2018 and 2023

To better characterise the rising incidence of *T. tonsurans* in *tinea corporis* and *tinea capitis* cases, the age and gender distribution of these patients were analysed in detail.

**FIGURE 4:**
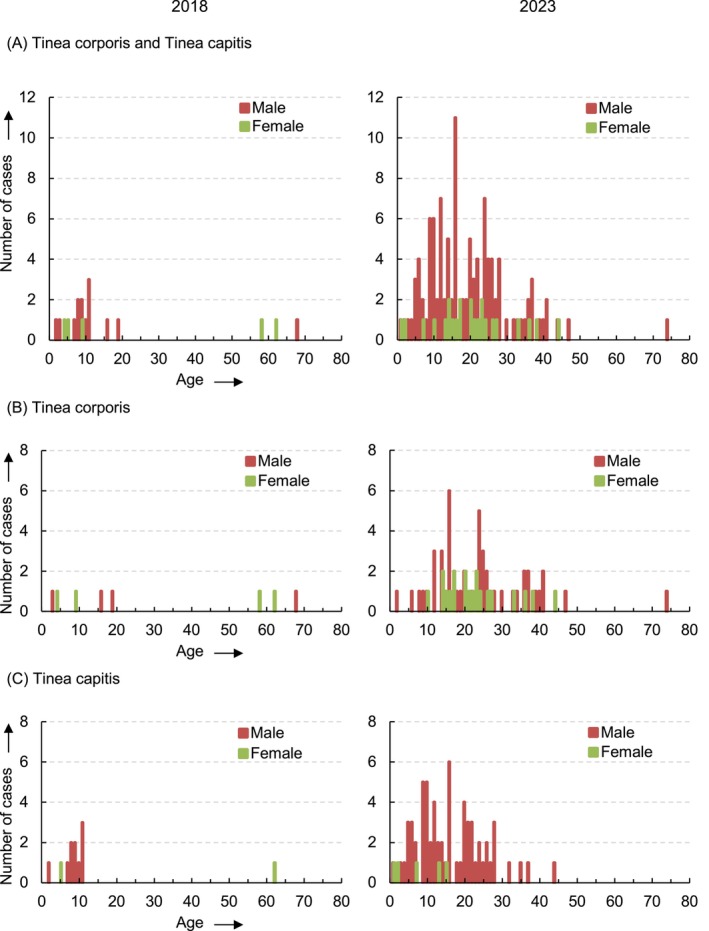
Distribution of patients with *T. tonsurans*‐induced *tinea corporis* and *tinea capitis* by gender and age in 2018 and 2023. Number of patients with a *T. tonsurans‐*positive culture segmented by gender and age for 2018 (left) and 2023 (right) for *tinea corporis* plus *tinea capitis* (A), *tinea corporis* alone (B) and *tinea capitis* alone (C).

Looking at the combined analysis of *tinea corporis* plus *tinea capitis* cases caused by *T. tonsurans* in 2018, a concentration of cases (73.3%; 14/19) was observed in children aged 2–11 years. The median age was 9 (IQR 6) years. Males were more frequently affected (73.7%; 14/19) than females (Figure 4A, left).

By 2023, the age distribution shifted, with most cases occurring in patients aged 5–37 years (88.8%; 119/134). The median age rose to 18 (IQR 13) years. The imbalance between genders was more profound compared to 2018, with men representing 82.1% (110/134) of cases (Figure 4A, right).

This pattern in age and gender distribution was also seen when the types of infection were analysed separately. However, the higher proportion of male patients was more pronounced in the *tinea capitis* group, with 92.7% (64/69), compared to 70.5% (48/68) in the *tinea corporis* group (Figure 4B,C).

## 4 Discussion

The aim of the study presented here was to assess the epidemiological profile of dermatophytes in Germany, focusing on changes in recent years. *T. rubrum* was the overall leading dermatophyte, and *tinea pedis* and *tinea unguium* were the most prevalent clinical types of infection. An increase in *tinea capitis* and *tinea corporis* was observed, with *T. tonsurans* emerging as the prevailing agent in *tinea capitis* and the second most common in *tinea corporis*. This new dominance of *T. tonsurans* was a consistent phenomenon in all three study centres across southern Germany, with young male patients being the most affected patient group.

The total study cohort comprised 1915 patients with dermatophyte‐positive cultures, with Tübingen as the largest of the three study centres. Potential confounding effects of the global COVID‐19 pandemic, which lasted from 2019 to 2022, were avoided by selecting the respective years. Hence, this study provides a robust dataset to explore the epidemiological landscape of dermatophytes in Germany.

Overall, *T. rubrum* was by far the most frequent dermatophyte, with *tinea pedis* and *tinea unguium* being the most common types of infection. Kromer et al., who analysed mycological culture results in three hospitals in central Germany from 2014 to 2016, found *tinea unguium* (50.8%) to be more frequent than *tinea pedis* (34.6%), but proportions for *tinea corporis*, *manus*, *capitis* and *faciei* were similar to the 2018 results presented here [7]. Equally, Ziegler et al. reported that *tinea capitis* accounted for 3.4% of all culture‐confirmed dermatophytoses from 1990 to 2014, matching the here observed 4.3% in 2018 [6]. However, comparing 2018 and 2023, a relative and absolute increase of *tinea capitis*, which doubled, and *tinea corporis* was observed. *Tinea capitis* is highly contagious, requires simultaneous topical and systemic treatment, and scarring with subsequent alopecia is feared. Consequently, there is a great economic and social interest in keeping overall infection rates low [18, 19].

Analysing certain types of infection regarding the distribution of pathogens, *tinea pedis* and *tinea unguium*, were caused in about 86%–90% by *T. rubrum*, followed by *T. interdigitale* and *T. mentagrophytes*, with no significant changes from 2018 to 2023. This was similarly seen by Kromer et al. and matches global data [3, 7]. The predominance of *T. rubrum* developed in the late 20th century and is suspected to be linked to increased urbanisation and usage of communal sports facilities [20]. Nevertheless, in restricted groups/areas, for example naval cadets, *T. interdigitale* or *T. mentagrophytes* may prevail [21, 22, 23]. In *tinea corporis*, the dominance of *T. rubrum* seen in central Germany from 2014 to 2016 and in the 2018 data presented in this study dipped considerably, with *T. tonsurans* emerging as the second most common dermatophyte by 2023 [7]. Also worldwide, *T. rubrum* is the most frequent causative agent of *tinea corporis* succeeded by the *T. mentagrophytes* complex, *M. canis* , *T. tonsurans* and *T. benhamiae* [3]. Interestingly, *T. benhamiae*‐associated infections, which were reported in 2014 to be on the rise in Germany [24], appear to be declining. While *T. benhamiae* caused 11.9% of *tinea corporis* cases in the analysis by Kromer et al. (2014–2016) [7], and still 7.0% of *tinea corporis* cases in 2018, its proportion shrank to 1.9% in 2023. According to the literature, the pathogen spectrum of *tinea capitis* in Germany has always been rather diverse. For example, in Würzburg from 7/2002 to 12/2014, Ziegler et al. observed *M. canis* (45.5%), *T. interdigitale* (20.0%) and *T. tonsurans* (9.1%) as the main dermatophytes, while Kromer et al. found *M. canis* , *T. mentagrophytes* and *T. benhamiae* to be the most frequent ones [6, 7]. In the 2018 data reported in this study, *T. tonsurans*, *M. canis* , *T. violaceum* and *T. soudanense* were the leading fungal agents, with *T. tonsurans* being responsible for two‐thirds of all cases by 2023. This predominance of *T. tonsurans* is seen in a couple of regions worldwide [4], but is especially true for the United Kingdom [25], the United States [26, 27] and the Caribbean [28]. In France, an increase of *T. tonsurans* in *tinea capitis* has been described by independent groups who analysed the time periods 2010–2015 as well as 2014–2016 and 2017–2019. They suggest the rise is linked to the high prevalence of *T. tonsurans* in the French Caribbean territories [29, 30]. In Germany, local outbreaks have so far been the focus [12, 13, 14]. However, in the study presented here, an increase in *T. tonsurans*‐associated infections was observed across all three study centres in southern Germany, which rather indicates a general change in the epidemiology of dermatophyte infections. *T. tonsurans* was not identified at all in Tübingen in 2018, yet it already accounted for 23.4% of all *tinea corporis* plus *tinea capitis* cases in Freiburg during the same period. This observation underscores the importance of multiple study centres in reliably identifying true epidemiological trends, especially when the sample size for each pathogen is limited.

*T. tonsurans* is traditionally known as the main causative agent of *tinea gladiatorum*, a dermatophytosis affecting combat sport athletes, particularly wrestlers [31, 32]. Here, the fungus is primarily transmitted from person to person, including asymptomatic carriers. In wrestling, athletes have close skin‐to‐skin contact, and concomitant skin trauma may facilitate infection. The often‐suggested transmission via inanimate objects, such as wrestling mats, seems debatable. A number of studies found wrestling mats to be contaminated with *T. tonsurans* [33], but whether these amounts were enough to cause new infections in athletes and/or whether regular disinfection of mats reduces the risk of transmission has not yet been sufficiently investigated [34]. Nevertheless, transmission of *T. tonsurans* via shared shavers and visits in barber shops/hairdressers, respectively, has repeatedly been suggested—in a French high‐level judo team [35] as well as in an Argentinean [36], Italian [37], German [38] and two Spanish [39, 40] case series. In the two very recent European case series reporting *T. tonsurans* infections of the head in association with barber shop visits from Bascon et al. and Addari et al., almost exclusively male patients with a relatively high (23.5 and 19.7 years) median age were described [37, 39]. Also, in the here presented analysis comparing *T. tonsurans*‐positive patients of 2018 and 2023, the median age and the male predominance increased.

*Tinea capitis* is typically diagnosed between the ages of 6 months and 10–12 years, with incidence decreasing as the composition of sebum changes at the onset of puberty [41]. The here observed shift in age and gender in patients with *T. tonsurans* infections fits well to the hypothesis of transmission in barber shops. Male adolescents/young adults very frequently go to barber shops and get currently popular haircuts with shaved temporal and occipital areas [39]. Findings of growing *T. tonsurans* in a barber shop, particularly on shavers, underline this hypothesis [17]. Therefore, intensified hygiene measures may be an important step towards breaking chains of infections.

Individual treatment of *tinea capitis* is usually performed empirically at the beginning and should be based on the most probable causative agent [18]. Itraconazole is the first choice for *Microsporum* and/or *Nannizzia* species and terbinafine for *Trichophyton* species [18]. Since *T. tonsurans* was identified here as the most common pathogen, it should be discussed to routinely use terbinafine in (southern) Germany. Furthermore, standardised and regular reporting on pathogen spectra at national level should be implemented to facilitate the early detection of changes.

A main limitation of this study is that only demographic, but no further characteristics of infected patients such as sources of infection were assessed. However, asking for specific information in the medical history is subject to recall bias. Secondly, there may be a selection bias, as patients referred to a university hospital might be more complex and/or severely affected and may not represent the general population. A third limitation is that our three study centres are all located in southern Germany, possibly not reflecting other parts of the country. Nevertheless, the three centres are large university hospitals with great catchment areas and are between 150 and 350 km apart.

## 5 Conclusions

In summary, this study shows an increase of *tinea capitis* and *tinea corporis* cases from 2018 to 2023, with *T. tonsurans* emerging as the most and second most common pathogen, respectively. Patients with *T. tonsurans* infections tended to be older, with a profound predominance of the male sex in 2023 compared to 2018. Since *tinea capitis* is complex to treat and may lead to irreversible alopecia, it is important to report such trends and initiate countermeasures. The increased median age and male dominance of affected people may support the hypothesis of barbershop visit‐associated infections; therefore, intensified screening and hygiene measures could be advisable. In addition, initial empiric treatment of *tinea capitis* should be adapted to the changed dermatophyte spectrum and consequently be performed with terbinafine.

## Author Contributions

**Julia Felicitas Pilz:** conceptualization, data curation, formal analysis, investigation, methodology, project administration, visualization, writing – original draft, writing – review and editing. **Martin Schaller:** data curation, methodology, project administration, writing – original draft, writing – review and editing. **Martin Köberle:** data curation, formal analysis, methodology, writing – original draft. **Alexandra Lorz:** data curation, investigation, writing – original draft. **Avend Bamarni:** data curation, investigation, writing – original draft. **Sebastian Sitaru:** methodology, data curation, writing – original draft. **Franziska Schauer:** conceptualization, data curation, investigation, project administration, writing – original draft. **Hans Peter Seidl:** investigation, writing – original draft. **Tilo Biedermann:** writing – original draft, funding acquisition, supervision, resources. **Alexander Zink:** conceptualization, funding acquisition, resources, supervision, writing – original draft. **Kilian Eyerich:** funding acquisition, resources, supervision, writing – original draft, project administration. **Anna Caroline Pilz:** writing – original draft, supervision, conceptualization, investigation, methodology, project administration, visualization, writing – review and editing.

## Conflicts of Interest

J.F.P. has no conflicts of interest to declare. M.S. is one of the three Editors in Chief of the journal Mycoses. M.K. has no conflicts of interest to declare. A.L. has no conflicts of interest to declare. A.B. has no conflicts of interest to declare. S.S. has no conflicts of interest to declare. F.S. has no conflicts of interest to declare. H.P.S. has no conflicts of interest to declare. A.Z. has no conflicts of interest to declare. T.B. has no conflicts of interest to declare. K.E. has no conflicts of interest to declare. A.C.P. has no conflicts of interest to declare.

## Supporting information


Tables S1–S3

## Data Availability

The data that support the findings of this study are available from the corresponding author upon reasonable request.
